# Pharmacological Profiling of *Calotropis Procera* and *Rhazya Stricta*: Unraveling the Antibacterial and Anti-Cancer Potential of Chemically Active Metabolites

**DOI:** 10.7150/jca.96848

**Published:** 2025-01-01

**Authors:** Sahar S. Alghamdi, Allulu Y. Alturki, Rizwan Ali, Rasha S. Suliman, Afrah E. Mohammed, Atheer Al Dairem, Zeyad I. Alehaideb, Raghad A. Alshafi, Sara A. Alghashem, Ishrat Rahman

**Affiliations:** 1 College of Pharmacy (COP), King Saud bin Abdulaziz University for Health Sciences (KSAU-HS), Riyadh, Kingdom of Saudi Arabia.; 2 Medical Research Core Facility and Platforms, King Abdullah International Medical Research Center (KAIMRC), Ministry of National Guard Health Affairs, Riyadh, Saudi Arabia.; 3 King Abdulaziz Medical City, Ministry of the National Guard - Health Affairs, Riyadh 11426, Saudi Arabia.; 4 Pharmacy department, Fatima College of Health Sciences (FCHS), Abu Dhabi, United Arab Emirates.; 5 Department of Biology, College of Science, Princess Nourah bint Abdulrahman University (PNU), P.O. Box 84428, Riyadh 11671, Saudi Arabia.; 6 Department of Basic Dental Sciences, College of Dentistry, Princess Nourah bint Abdulrahman University (PNU), P.O. Box 84428, Riyadh 11671, Saudi Arabia.; 7 Microbiology and Immunology Unit, Natural and Health Sciences Research Center, Princess Nourah bint Abdulrahman University, Riyadh, Saudi Arabia.

**Keywords:** Anti-bacterial, Anti-cancer, *Calotropis procera, Rhazya stricta*, Apocynaceae*, In silico*

## Abstract

**Background:** The increasing prevalence of cancer and bacterial resistance necessitates more effective anti-cancer and anti-bacterial treatments. This study explores the potential of medicinal plants, specifically *Calotropis procera* (*C. procera*) and *Rhazya stricta* (*R. stricta*), in addressing this need, aiming to uncover new therapeutic interventions.

**Methods:** Various extraction methods for the leaves of *C. procera* and *R. stricta* were employed to investigate the anti-bacterial and anti-cancer properties of these herbs. For anti-bacterial testing, extracts were prepared using water, chloroform, and ethyl acetate, and their activity against methicillin-resistant *Staphylococcus aureus* (*S. aureus*) (MRSA) and *Escherichia coli* (*E. coli*) was assessed. The anti-cancer potential was evaluated through MTT cytotoxicity assays on various cancer cell lines and further testing using high-content imaging (HCI)-Apoptosis Assay and the ApoTox-GloTM Triplex Assay. Liquid chromatography-mass spectrometry (LC-MS) was used to identify the secondary metabolites of *C. procera*, and computational predictions were performed to assess the activity of these metabolites.

**Results:** The leaf extracts of both *C. procera* and *R. stricta* demonstrated antibacterial activity against *S. aureus* and *E. coli*. The *C. procera* ethyl acetate extract exhibited potent anti-cancer effects on several cancer cell lines. The research also revealed a dose-dependent induction of apoptosis and a decline in cell viability. Computational predictions suggested the identified metabolites were active as nuclear receptor ligands and enzyme inhibitors, with good oral bioavailability. Most metabolites were found to be immunologic and cytotoxic, except for proceragenin and calotropone, which were determined to be non-cardiotoxic.

**Conclusion:** The study's findings demonstrate the remarkable cytotoxic and antibacterial effects of *C. procera* extracts prepared using ethyl acetate. These results pave the way for further *in vitro* studies to explore the full potential of these extracts and highlight the presence of chemically active metabolites in *C. procera*, which hold promise as lead molecules for the development of novel therapies targeting bacterial infections and cancer while minimizing potential side effects.

## Introduction

Natural products, including medicinal plants, have gained significant attention in recent years due to their potential in treating various diseases, such as infectious diseases and cancer 1, 2. The rise of antibiotic resistance poses a serious threat to global public health 3. Pathogens are becoming increasingly resistant to antibiotics, affecting both individuals and healthcare systems. Given the increasing prevalence of antibiotic resistance among pathogenic bacteria, there is a critical need to discover and develop alternative antimicrobial agents. Therefore, assessing the antimicrobial activity of natural products such as plant extracts is crucial for identifying potential sources of new antimicrobial compounds. Additionally, chemotherapy and radiotherapy are the main treatment choices for cancer. Surgical removal and careful assessment of the individual's medical condition, along with the potential benefits and risks of the procedure, are the preferred approaches for managing localized cancer 4, 5. The continuous advancements in cancer research, prevention strategies, and therapies have greatly contributed to reducing the cancer burden 6. One of the approaches to overcome these challenges is the use of natural products that could possess several bioactive metabolites and exhibit dual anti-bacterial and anti-cancer activities.

Medicinal plants such as *C. procera* and *R. stricta* have shown promising biological activities such as antioxidant, anti-inflammatory, and anti-tumor properties, making them attractive for the discovery of new bioactive agents 7, 8. *C. procera* is a wild species belonging to the* Apocynaceae* family, which is found across West Africa to Southeast Asia. It is commonly known as milkweed due to its distinctive white and sticky latex exudation from all parts of the plant, making it an intriguing subject for phytochemical studies 9. The presence of active phytoconstituents such as cardiac glycosides, flavonoids, and triterpenes in *C. procera* has been reported in the literature, highlighting its potential biological activities 10, 11. In a previously reported study, various extracts of *C. procera* were tested against a range of bacteria including *Streptococcus agalactiae*, *Escherichia coli, Klebsiella pneumonia, Salmonella typhi, Serratia marcescens,* and* Staphylococcus aureus*. The results revealed that *C. procera*, along with its endophytic bacteria *B. siamensis*, exhibited a broad-spectrum antibacterial effect 12. Furthermore, *C. procera* was found to contain a secondary metabolite named Uzarigenine, which demonstrated moderate cytotoxic activity against human cancer cell lines such as colorectal adenocarcinoma (HT29), hepatocellular carcinoma (HepG2), and the mouse fibroblast cell line (NIH-3T) 13.

Another noteworthy species from the *Apocynaceae* family is *R. stricta*, which is widely distributed in South Asia and the Middle East 14. *R. stricta* is recognized as a rich source of alkaloids 15, 16. One of its active components, Strictanol, has been found to exhibit activity against *E. coli* and *Pseudomonas aeruginosa*. Additionally, Tetrahydrosecamine, another active compound from *R. stricta*, has shown broad-spectrum antimicrobial activity. Moreover, *R. stricta*, has demonstrated excellent antioxidant and antiproliferative activities, making it a potential candidate for antitumor therapy, particularly in hepatocellular carcinoma treatment. In recent studies, Isopicrinine, a newly identified alkaloid from *R. stricta*, has shown promising results as an anti-cancer treatment against breast cancer cell lines 17. Its mechanism of action involves the downregulation of survivin, a protein associated with cancer cell survival 18.

Thus, our study aims to determine the most optimal solvent for the extraction process of two medicinal herbs, *C. procera* and *R. stricta* that would confer the extracts with biological anti-bacterial and anti-cancer activities. Furthermore, identify the secondary metabolites present in the extracts and evaluate their pharmacokinetic, molecular targets, safety properties, and feasibility for further research in the drug development process.

## Materials and Methods

### Chemical Extraction and Identification of Metabolites

#### Chemicals and Reagents

Dulbecco's Modified Eagle Medium (DMEM) plus GlutaMax-1 (4.5g/l D-Glucose, 25 mM HEPES, Pyruvate), fetal bovine serum (FBS), TrypLE™ Express, and Dulbecco's phosphate-buffered saline (PBS) were provided by Gibco® (Waltham, MA). NP-40 cell lysis buffer was purchased from Thermo Fisher Invitrogen (Carlsbad, CA). Methanol and ethanol were obtained from Honeywell Riedel-de Haen (Seelze, Germany) and Merck (Kenilworth, NJ). Dichloromethane and chloroform were obtained from Sigma-Aldrich (St. Louis, MO). Dimethyl sulfoxide (DMSO) was purchased from Calbiochem (San Diego, CA). The solvents used were chromatography-grade or equivalent. Purified carbon dioxide (CO_2_) gas was provided by Saudi Industrial Gas (Dammam, Saudi Arabia). Ultra-pure water was produced using a Millipore (Billerica, MA) system with a resistivity reading of 18.2 MΩ·cm at 25°C.

#### Collection and Authentication of *C. procera* and *R. stricta*

*C. procera* and *R. stricta* leaves were wildcrafted around the Riyadh regional area, the Kingdom of Saudi Arabia. In our study, we have followed the IUCN Policy statement on research involving species at risk of extinction and the convention on the trade in endangered species of wild fauna and flora, in addition to the authentication by Professor Mona AlWhibi of Botany and Taxonomy at King Saud University. The leave parts of *C. procera* and *R. stricta* were rinsed with filtered water and left to dry under a stream of slightly warm dry air. The leave parts were finely powdered with an electric motor grinder and kept at room temperature in the dark until extraction. A voucher sample was deposited at King Saud University (Herbium. Department of Botany and Microbiology) with accession numbers 24317 and 24321, respectively.

#### Extraction of the Plants

In this study, 500.0 mg of dried *C. procera* and *R. stricta* were extracted using 10.0 mL of high-purity ethanol, ethyl acetate, chloroform, and water under high-power sonication using a Sonics (Newton, USA) Vibra-Cell Ultrasonic Liquid Processor (Model GEX-130 probe-sonicator) for 90 mins. The sonicated extract was filtered using a Sartorius stadium biotech (Göttingen, Germany) quantitative ashless filter paper under gravity flow and dried in an incubator set at 40.0˚C. The remaining dried pellet residue was weighted and diluted with 100.0 to 300.0 µL of DMSO by vortex until completely dissolved. The reconstituted extract was stored at a cool temperature in the dark until further use. The average extraction yield is a measure of the final product amount (in mg) at the end of the extraction process, expressed as a percentage of the volume of starting material used. The percentage of the *C. procera* for the leaves-derived ethanol, ethyl acetate, chloroform, and water solvent extracts were (%) 20.34 ± 0.20, 4.93 ± 0.01, 8.85 ± 0.07, and 16.26 ± 0.13, respectively. The average extraction yield of the *R. stricta* for the leaves-derived ethanol, ethyl acetate, chloroform, and water solvent extracts were (%) 24.32 ± 0.08, 8.21 ± 0.01, 16.89 ± 0.08, and 30.06 ± 0.02, respectively.

### Biological Testing

#### Anti-bacterial Screening

In this study, we assessed the antimicrobial susceptibility of the water, chloroform, and ethyl acetate extracts of *C. procera* and *R. stricta* against a range of bacteria, including Gram-positive methicillin-resistant *Staphylococcus aureus* (*S. aureus*) (MRSA) and Gram-negative *Escherichia coli* (*E. coli*). The antimicrobial susceptibility testing (AST) was conducted using the agar well diffusion assay. The bacterial strains used in the study were sourced from the Bio-house Medical Laboratory in Riyadh, Saudi Arabia. The antimicrobial testing was undertaken to evaluate the potential of the extracts from *C. procera* and *R. stricta* as sources of novel antimicrobial compounds.

Each strain's pure cultures were subcultured on nutritional agar medium (Oxoid) and grown for 24 hours at 37°C. Direct colonies from each culture were used for 0.5 McFarland standard bacterial solutions (1.5 x 10^8^ CFU/mL) made in saline. For the bactericidal test, the plant material underwent extraction using various solvents, followed by drying. The antimicrobial test involved using the dried samples (for each extract in a different solvent), each weighing 1 mg, which were then dissolved in 1 ml of DMSO. Subsequently, 40 microliters of each extract were added to individual wells created in the agar plates. The plates were then stored for an hour under aseptic conditions to facilitate the easy diffusion of the tested substance in the agar before inoculating the plates with the tested microorganisms. Ampicillin was used as a positive control, and distilled water (DW) served as a negative control. According to the Clinical and Laboratory Standards Institute, the temperature of the plates was maintained for 24 hours at 37 °C. The inhibition zone, which represented the clean area around each well, was measured in millimeters and served as a visual representation of the effectiveness of the tested drugs. Four replicates were performed for each test.

#### Cell Proliferation Assay (MTT)

The anticancer activity of the leave extracts of *C. procera* and *R. stricta* in ethanol, ethyl acetate, chloroform, and water were investigated against four distinct cancer cell lines, including liver cancer (HepG2), colorectal cancer (HCT8), and breast cancer (MDA-MB-231). These cell lines were procured from the American Type Culture Collection (ATCC) in the United States. Additionally, an isolated and characterized breast cancer cell line (KAIMRC2) obtained from the King Abdullah International Medical Research Center (KAIMRC) in Riyadh, Saudi Arabia, was also included in the study, as previously characterized and reported 19. The objective of this experiment was to identify the extract and solvent combination that demonstrates the most potent anticancer activity against specific cancer cell lines. This information is crucial for selecting the most promising candidates for further in-depth investigations.

A 96-well plate was first filled with 100 µL of growth media containing 5 x 10^3^ cells per well. Following a 24-hour incubation period, the cells were treated with various doses of each extract, ranging from 0 to 500 µg/mL. The use of various doses of each extract, ranging from 0 to 500 µg/mL, allows for the assessment of the extract's effects across a range of concentrations. This approach helps in determining the extract's potential impact at different dosage levels, thereby providing valuable insights into its dose-dependent effects. After the 48-hour treatment, the cells were treated with 10 mg/mL of MTT solution. Following 3 hours at 37 °C of incubation, 100 µL of the DMSO solvent was added to each well. Plates were then covered in foil and shaken for 15 minutes on an orbital shaker. After 45 mins, the absorbance at 590 nm was finally measured and half-maximal inhibitory concentration (IC_50_) values for each extract were determined from the dose-response curve. A total of three replicates of each experiment were performed for each extract.

#### High Content Imaging (HCI)

Following the initial screening, the *C. procera* ethyl acetate extract exhibited the highest potency in HCT8 and KAIMRC2 cell lines. Consequently, further testing was conducted for these specific cell lines utilizing High Content Imaging (HCI) techniques which offers a powerful and comprehensive approach to assessing cellular morphology, subcellular structures, and various cellular processes at a high throughput and quantitative level. By employing HCI, it is possible to obtain detailed information regarding the effects of the *C. procera* ethyl acetate extract at the cellular and subcellular levels in the HCT8 and KAIMRC2 cell lines.

HCT8 and KAIMRC2 cells were seeded in a 96-well plate at 20,000 cells per well. The *C. procera* ethyl acetate extract, and the positive control drug Mitoxantrone was used due to their potential as an effective agent for the treatment of broad types of tumors and advanced breast cancer. Different treatments were exposed to the cells for 48 hours using four graded concentrations of the prepared extracts that were used in the treatment 31.25 µg/mL, 62.5 µg/mL, 125 µg/mL, and 250 µg/mL. The chosen concentrations cover a spectrum that facilitates the evaluation of the dose-response relationship of the ethyl acetate extract on the HCT8 and KAIMRC2 cell lines. This concentration range enables the examination of the cellular response as the extract dosage increases. The cells were stained with Invitrogen™ Calcein AM (2 µg/mL), HOECHST33342 (2.5 µg/mL), and propidium iodide (2.5 µg/mL) for 45 minutes at 37 °C and 5% CO_2_. Plates were scanned using a Molecular Devices ImageXpress® Microsystem, and the MetaXpress® software, Downingtown, PA, USA, was used to process the image data. The Cell Health module of the MetaXpress software was used to determine the percentage of viable live and dead cells. There were three replicates performed for each cell line.

#### ApoTox-Glo™ Triplex Assay

The ApoTox-Glo™ Triplex Assay is a comprehensive biological assay used to evaluate cell viability, cytotoxicity, and caspase 3/7 activation and cleavage. This assay involves the use of specific reagents containing substrates for measuring cell viability and cytotoxicity, as well as a luminescence-based reagent for assessing caspase 3/7 activity. The assay provides valuable insights into the effects of compounds on cell health, cytotoxicity, and apoptotic pathways through the measurement of fluorescence and luminescence signals. The initial evaluation of the *C. procera* ethyl acetate extract demonstrated the highest potency in HCT8 and KAIMRC2 cell lines. As a result, further testing of this extract in these cell lines is crucial to elucidate the specific mechanisms underlying the observed effects, thereby providing valuable insights into the extract's potential as an anticancer agent.

The Promega ApoTox-Glo™ triplex assay was conducted as directed by the Promega manufacturer. The cells were treated with various doses of *C. procera* ethyl acetate extract for 24 hours in 96 well plates. The viability and cytotoxicity reagents were then added to each well and briefly mixed on an orbital shaker. For 30 minutes, the plates were incubated at 37 °C. The fluorescence was subsequently measured using the Envision plate reader (Perkin Elmer) at the following two-wavelength sets: 400Ex/505nm (Viability) and 485Ex/520nm (Cytotoxicity). The luminescence associated with caspase 3/7 activation was examined with an assay to decide the degree of apoptosis. The plates were rapidly agitated on an orbital shaker (300 to 500 rpm for 30s) after the addition of the Caspase-Glo® 3/7 Reagent (100 µL/well) and then incubated for an additional 30 min at room temperature. The Luminescence was measured using a Perkin Elmer plate reader.

#### LC-ESI-QTOF-MS Chemical Characterization of Metabolites

The utilization of Liquid Chromatography-Electrospray Ionization-Quadrupole Time-of-Flight-Mass Spectrometry (LC-ESI-QTOF-MS) for the characterization of *C. procera* ethyl acetate metabolites is warranted due to its ability to provide comprehensive and tentative identification of the chemical constituents present in the extract. This advanced analytical technique allows for the tentative identification of a wide range of compounds, including phenolic compounds, flavonoids, and other bioactive molecules, which are crucial for understanding the chemical composition and bioactive properties of the extract. Furthermore, LC-ESI-QTOF-MS enables the elucidation of the chemical structures of metabolites through fragmentation patterns and accurate mass measurements, thereby facilitating the identification and characterization of the chemical entities present in the *C. procera* ethyl acetate extract. This comprehensive characterization is essential for gaining insights into the potential bioactive components responsible for the observed pharmacological activities. Additionally, the identification of metabolites using LC-ESI-QTOF-MS can provide essential information for further *in silico* studies contributing to the development of effective new therapeutic agents with potential anticancer and antibacterial properties derived from natural sources.

The analysis was performed on the Agilent1260 Infinity HPLC system (AGILENT, Germany) coupled with Agilent 6530 Quadrupole Time of Flight (AGILENT, Singapore). Separation was performed using Agilent Extend-C18 column (2.1 mm × 50 mm, 1.8 μm) with the following elution gradient; 0-1 min, 5% B; 1-11 min, 5-100% B; 11-13 min, 95%B; 13-15 min, 5%B; 15-16 min, 5%B using mobile phase A (0.1% HCOOH in water) and mobile phase B (0.1% HCOOH in Methanol). The injection volume was 10 µl, and the flow rate was set as 300 µl/min. The MS1 acquisition method was directly utilized for the acquisition of positive-ion and negative-ion Electrospray Ionization (ESI) mass spectra mode. The cone voltage was adjusted in the range between 25 and 50 V. Fifty scans from 100 to 600 Daltons were collected to generate the averaged spectra. The capillary voltage was optimized at 4.5 kV in positive ion mode and - 3.1 kV in negative ion mode. The mass spectrometer parameters were set as follows: Gas Temperature = 300 °C; Gas flow = 8 I/min; Nebulizer = 35 psig; Sheath Gas Temperature = 350 and Sheath Gas flow was 11. MS1 data was generated by Agilent Mass Hunter qualitative analysis software.

#### Statistical Analysis

The biological data are presented as the standard deviation of the means from three independent experiments (SD). To compare the two groups, an unpaired student t-test was used. The threshold for statistical significance level was established at p <0.05. Statistical analysis was performed using GraphPad Prism Software version 10 (SanDeigo, California, USA).

### *In Silico* Computational Analysis

#### Computational Analysis

The chemical molecules present in the *C. procera* ethyl acetate extract were identified using Liquid Chromatography-Electrospray Ionization-Quadrupole Time-of-Flight-Mass Spectrometry (LC-ESI-QTOF-MS), enabling the comprehensive characterization of metabolites. Subsequently, computational analysis was performed using the simplified molecular-input line-entry system (SMILES), and the 2D chemical structure of the identified metabolites was generated using ChemDraw software. Additionally, computational tools such as PASS online, Molinspiration, SwissADME, and ProTox-II web servers were employed to further analyze the identified chemical entities, facilitating the assessment of their pharmacological and toxicological properties.

This integrated approach involving experimental LC-ESI-QTOF-MS analysis and computational tools allows for the thorough characterization and evaluation of the chemical constituents present in the *C. procera* ethyl acetate extract. By leveraging these methodologies, it becomes possible to gain a deeper understanding of the extract's chemical composition, potential bioactivity, and safety profile, thereby laying the foundation for the rational exploration and development of its pharmacological applications, particularly in the context of anticancer and antibacterial research and drug discovery.

#### PASS Online

The Prediction of Activity Spectra for Substances (PASS) website was utilized to predict the anticancer and antibacterial activity based on the 2D chemical structures of the identified metabolites. The platform offers predictions for over 4000 types of biological activities. Importantly, the predicted biological activity profile for a compound can be obtained solely based on its structural formula and the PASS system employs Multilevel Neighborhoods of Atoms (MNA) structure descriptors to generate predictions, simultaneously forecasting 3678 types of activity with an average prediction accuracy of approximately 95% through leave-one-out cross-validation. In the context of predicted biological activity, the putative activity (P_a_) values exceeding 0.5 (Pa > 0.5) and putative inactivity (P_i_) values approaching zero are indicative of promising biological activity, as per the predictions obtained from the PASS platform (http://way2drug.com/PassOnline/) 20.

#### Molinspiration

The Molinspiration web server (https://www.molinspiration.com/) was employed to estimate the bioactivity score for metabolites against a range of biological targets, including G protein-coupled receptors (GPCR), ion channels, nuclear receptors, kinases, proteases, and enzymes. This tool aids in predicting the molecular targets for the identified metabolites, providing insight into their potential biological activities. The bioactivity score serves as an indicator of a molecule's activity, with a predicted score equal to or greater than 0.00 signifying activity, a score ranging from -0.50 to 0.00 indicating moderate activity, and a score less than -0.50 denoting inactivity against the specified targets.

#### Absorption, Distribution, Metabolism, and Excretion (ADME)

ADME properties were predicted using SwissADME (http://www.swissadme.ch/), a web tool developed and maintained by the Molecular Modeling Group of the (SIB) Swiss Institute of Bioinformatics. This website allows the computation of physicochemical descriptors and the estimation of ADME parameters (absorption, distribution, metabolism, and excretion), pharmacokinetic properties, drug-like nature, and medicinal chemistry friendliness of one or multiple small molecules, providing invaluable support for drug discovery efforts. This comprehensive platform serves as a valuable resource for evaluating the pharmacokinetic and medicinal chemistry characteristics of small molecules, thereby facilitating informed decision-making in drug design and development 21.

#### ProTox-II

Using the ProTox-II website (https://tox-new.charite.de/protox_II/), the toxicity prediction of the identified metabolites was conducted, encompassing a wide range of toxicity endpoints such as acute toxicity, hepatotoxicity, cytotoxicity, carcinogenicity, mutagenicity, immunotoxicity, and adverse outcomes (Tox21) pathways, along with toxicity targets. This comprehensive prediction was facilitated by the incorporation of molecular similarity, fragment propensities, most frequent features, and machine-learning models, amounting to a total of 33 models. The platform offers uncomplicated predictions of different levels of toxicities for the tested molecules 22.

#### Pred-hERG 5.0 II Webserver

Using the Pred-hERG 5.0 web application (http://predherg.labmol.com.br/), the identified metabolites were subjected to computational screening to assess their potential impact on the blockage of hERG K^+^ channels, which is associated with cardiac toxicity. The platform generates probability maps that illustrate the contribution of individual atoms to the blockage of the hERG K^+^ channels, categorizing the compounds as non-blockers, weak/moderate blockers, or strong blockers. In these maps, green denotes blockage, red indicates non-blockage, and grey signifies no blockage at all. This detailed assessment aids in understanding the potential cardiac effects of the identified metabolites, contributing to informed decision-making in drug development and safety evaluation 23.

#### Predictions of Endocrine Disruptome

The Endocrine Disruptome is an open-source prediction tool (http://endocrinedisruptome.ki.si/) designed to predict the binding of molecules to 14 nuclear receptors, which include androgen receptor (AR); estrogen receptors (ER) α and β; glucocorticoid receptor (GR); liver X receptors (LXR) α and β; mineralocorticoid receptor (MR); peroxisome proliferator-activated receptors (PPAR) α, β/δ, and γ; progesterone receptor (PR); retinoid X receptor (RXR) α; and thyroid receptors (TR) α and β. These receptors are docked using the Docking Interface for Target Systems (DoTS) technique, and the results are classified into four main categories based on the likelihood of the molecule binding to the receptor. The classifications are denoted by colors: red for a high likelihood of binding, orange/yellow for a medium likelihood of binding, and green for a low likelihood of binding 24.

## Results

### Anti-bacterial Activity of *R. stricta* and *C. procera*

The antibacterial activity of *R. stricta* and *C. procera* extracts against *E. coli* and *S. aureus* was evaluated using aqueous, chloroform, and ethyl acetate solvents. Both plant types demonstrated activity against *E. coli* and *S. aureus*, with the exception of the aqueous extract from *C. procera*, which exhibited no activity against *S. aureus*. The efficacy of the extracts varied based on the plant type and the extraction solvent used. Unfortunately, due to the unavailability of samples extracted with the ethanol solvent, the evaluation of ethanol fraction was not included in our analysis.

**Figure 1:**
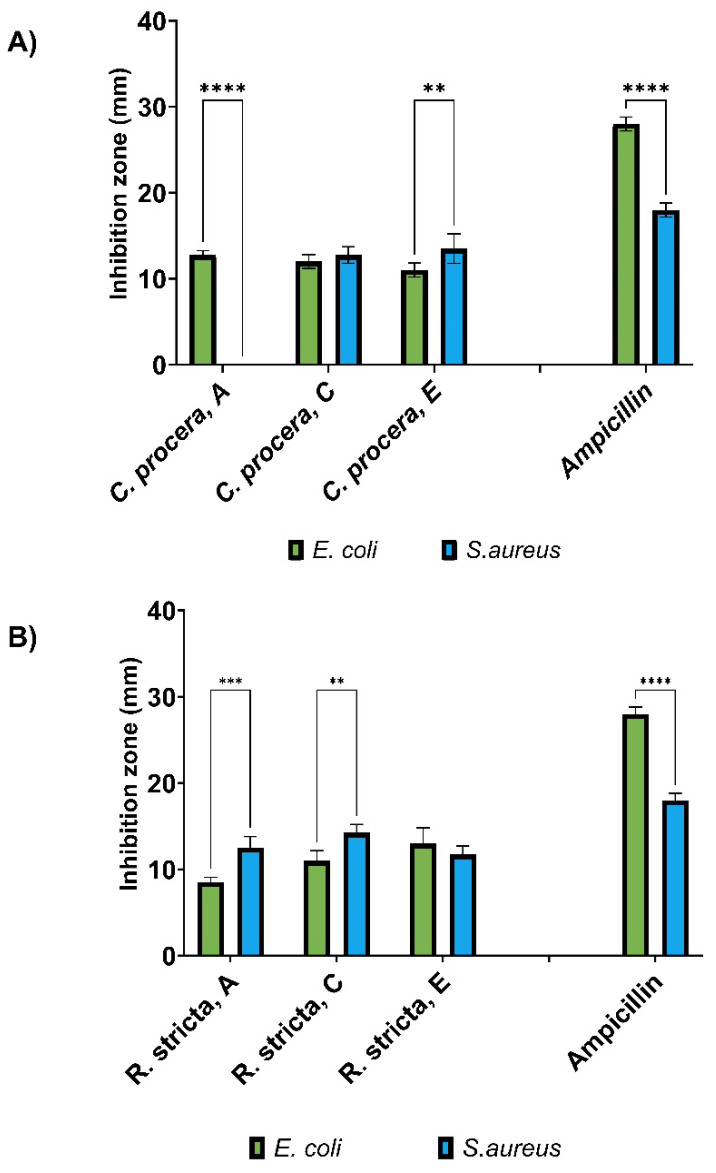
Antimicrobial activity of A) *C. procera* and B) *R. stricta* extracts using the agar well diffusion assay. Inhibition zones (mm) were determined for water (A), chloroform (C), and ethyl acetate (E) extracts of each plant/extract. Ampicillin served as a positive control, and the experiments were conducted with four replicates (n=4). The statistical significance of ^**^ is indicated by a p-value of 0.001, the significance of ^***^ is indicated by a p-value of 0.0001, while the significance of ^****^ is denoted by a p-value of less than 0.0001,

Chloroform extraction of *R. stricta* displayed the highest activity against *S. aureus*, as evidenced by an inhibition zone of 14.3 ± 0.9 mm, while ethyl acetate extraction of *C. procera* demonstrated the highest activity against *S. aureus*, with an inhibition zone of 13.5 ± 1.7 mm as shown in **Figure 1**. In comparison, the positive control Ampicillin exhibited activity with inhibition zones of 28 ± 0.9 mm and 18 ± 0.8 mm against *E. coli* and *S. aureus*, respectively. The activity of all extracts surpassed 50% of Ampicillin's activity against *S. aureus* and was less than 50% of its activity against *E. coli*. Statistical analysis revealed significant differences in the antibacterial activity of the tested extracts, and bacterial responses, emphasizing the diverse and noteworthy outcomes obtained from the assessment of the antibacterial activities of the extracts.

### Cell Viability and Proliferation Analysis

Upon conducting the MTT assay, the cytotoxic effects of *C. procera* and *R. stricta* extracts (dissolved in different solvents) were evaluated on various cancer cell lines including HepG2, HCT8, MDA-MB-231, and KAIMRC2. The screening of multiple cancer cell lines was performed to comprehensively assess the cytotoxic activity of the extracts across different cancer types, providing a broader understanding of their potential therapeutic applicability and enabling a comprehensive screening of their cytotoxic effects.

**Figure 2:**
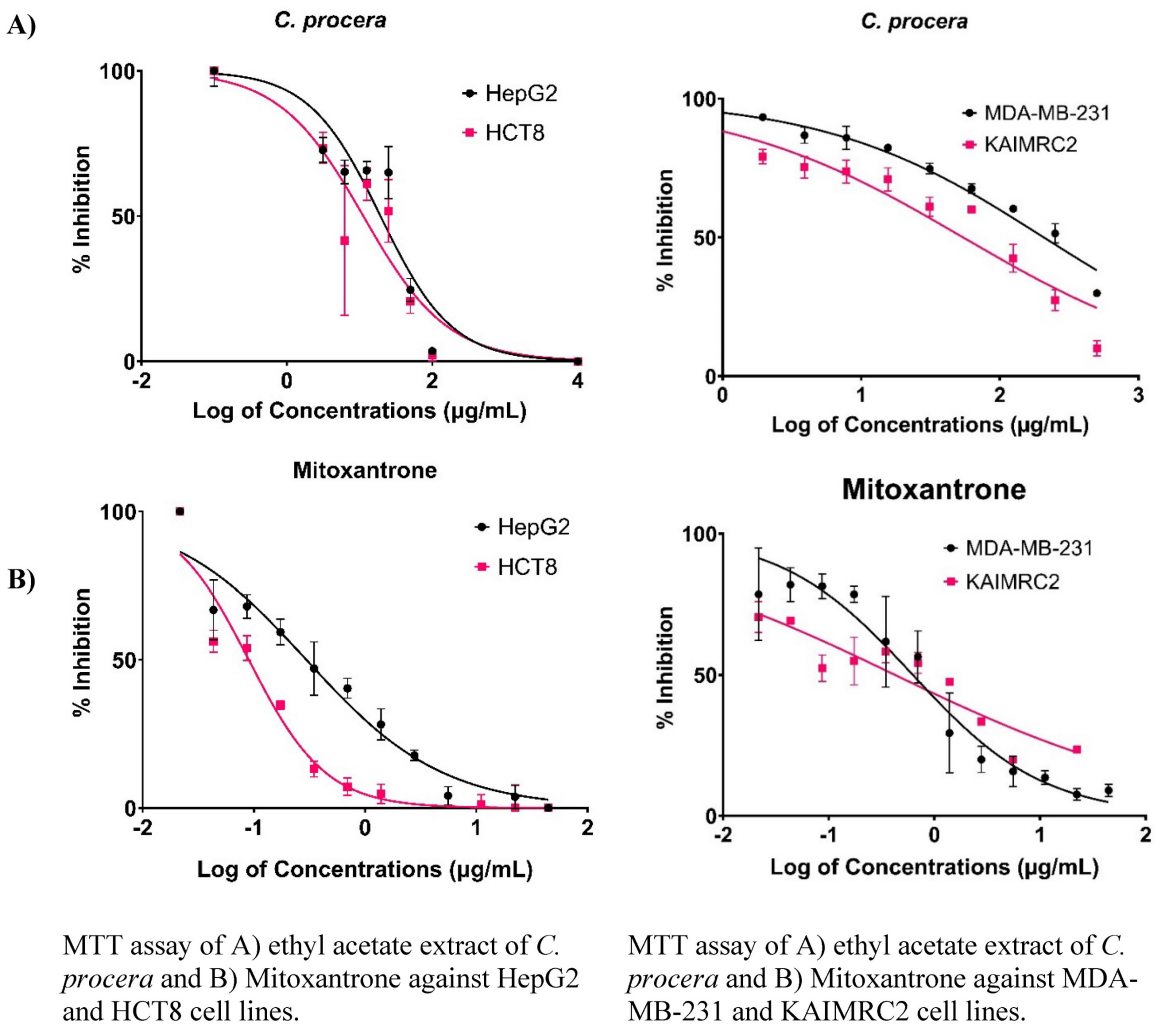
MTT Assay Results for *C. procera* samples Aganis Several Cancer Cell Lines.

As summarized in **Table 1, and Figure 2**, the ethyl acetate *C. procera* extract showed sensitivity against HepG2 (IC_50_ of 194.0 µg/mL) and HCT8 (IC_50_ of 112.6 µg/mL) cell lines. Since the ethyl acetate *C. procera* had the lowest IC_50_ among the tested extracts, further tests with this extract on KAIMRC2 and MDA-MB-231 cell lines revealed IC_50_ values of 207.4 and 54.37 µg/mL, respectively (**Table 1, and Figure 2**). These findings demonstrate the potential cytotoxic effects of the *C. procera* extract on various cancer cell lines, as supported by the tabulated IC_50_ values for different extracts against cancer cell lines, providing a comprehensive understanding of their cytotoxic activities. It is important to note that *R. stricta* extracts exhibited lower potency compared to *C. procera* extracts, which constrained further investigations on *R. stricta* extracts.

### Cytotoxicity Evaluation of Ethyl Acetate Extract of *C. procera* Using HCI Apoptosis Assay

**Figure 3:**
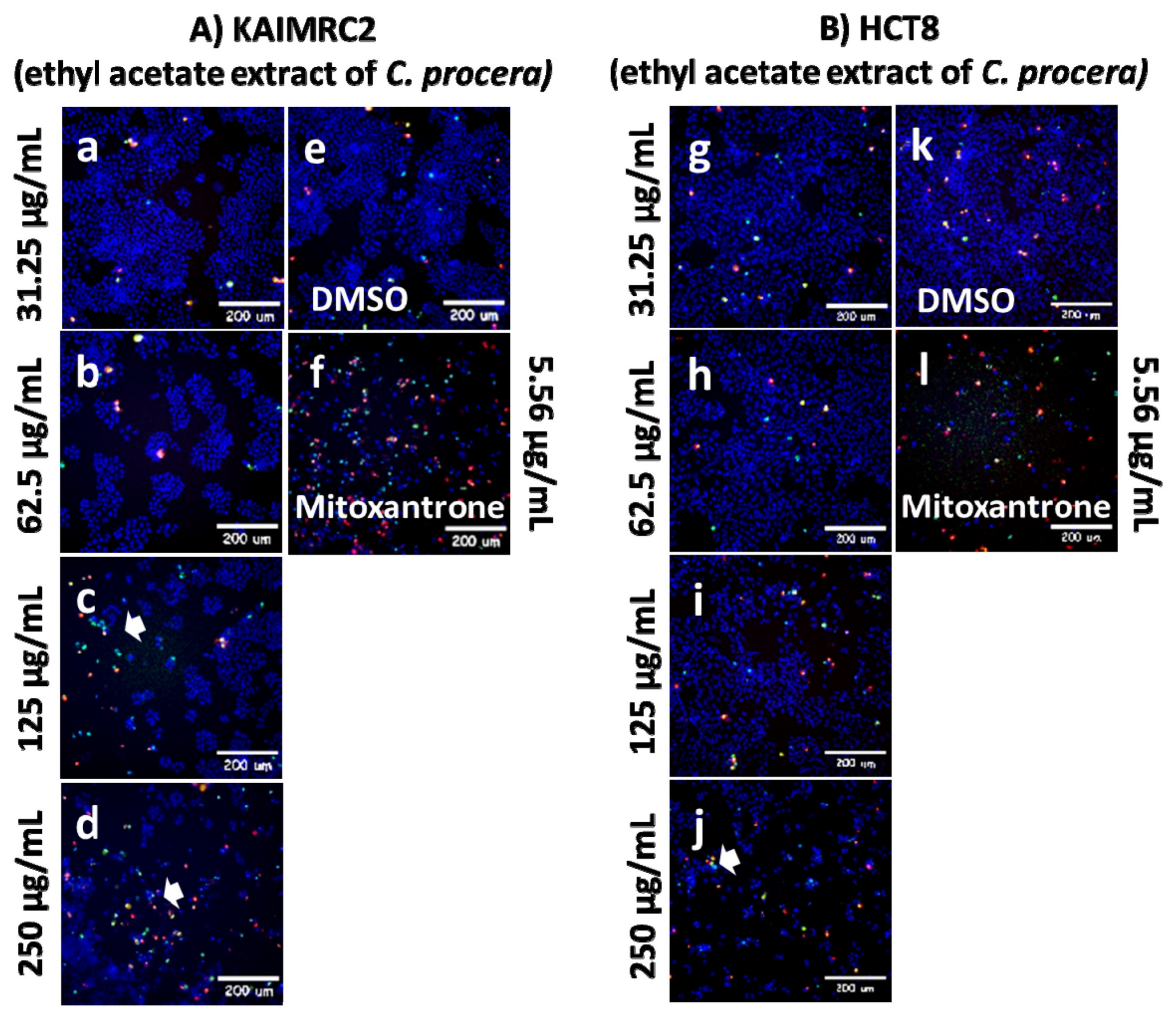
HCl Apoptosis Assay of Ethyl Acetate extract of *C. procera* against A) KAIMRC2 and B) HCT8 cell lines.

The ethyl acetate extract of *C. procera* demonstrated the highest and most potent anti-cancer activity in the MTT cytotoxic assay among all the tested extracts, leading to further testing exclusively with the ethyl acetate *C. procera* extract using HCI. The study focused on treating the breast cancer cell line, KAIMRC2, and the colorectal cancer cell line, HCT8, with increasing concentrations of the *C. procera* ethyl acetate extract as shown in **Figure 3**. The post-treatment cells were then stained using HOECHST 33342, propidium iodide (PI), and YoPro-1™. The HOECHST 33342 stain highlighted the nucleus in blue, the PI stain indicated dead cells in red, and the YoPro-1 stain identified apoptotic cells in green.

The KAIMRC2 and HCT8 cell lines were subjected to varying concentrations of the ethyl acetate extract of *C. procera*. Notably, distinct variations were observed between the two cell lines, particularly when treated with higher extract concentrations of 125 µg/mL and 250 µg/mL. It was observed that KAIMRC2 cells exhibited a higher tendency for apoptosis compared to HCT8 cells under these concentrations. This suggests that the ethyl acetate extract of *C. procera* may possess a more profound anti-cancer effect on the KAIMRC2 cell line.

### ApoTox-Glo Triplex Assay

**Figure 4:**
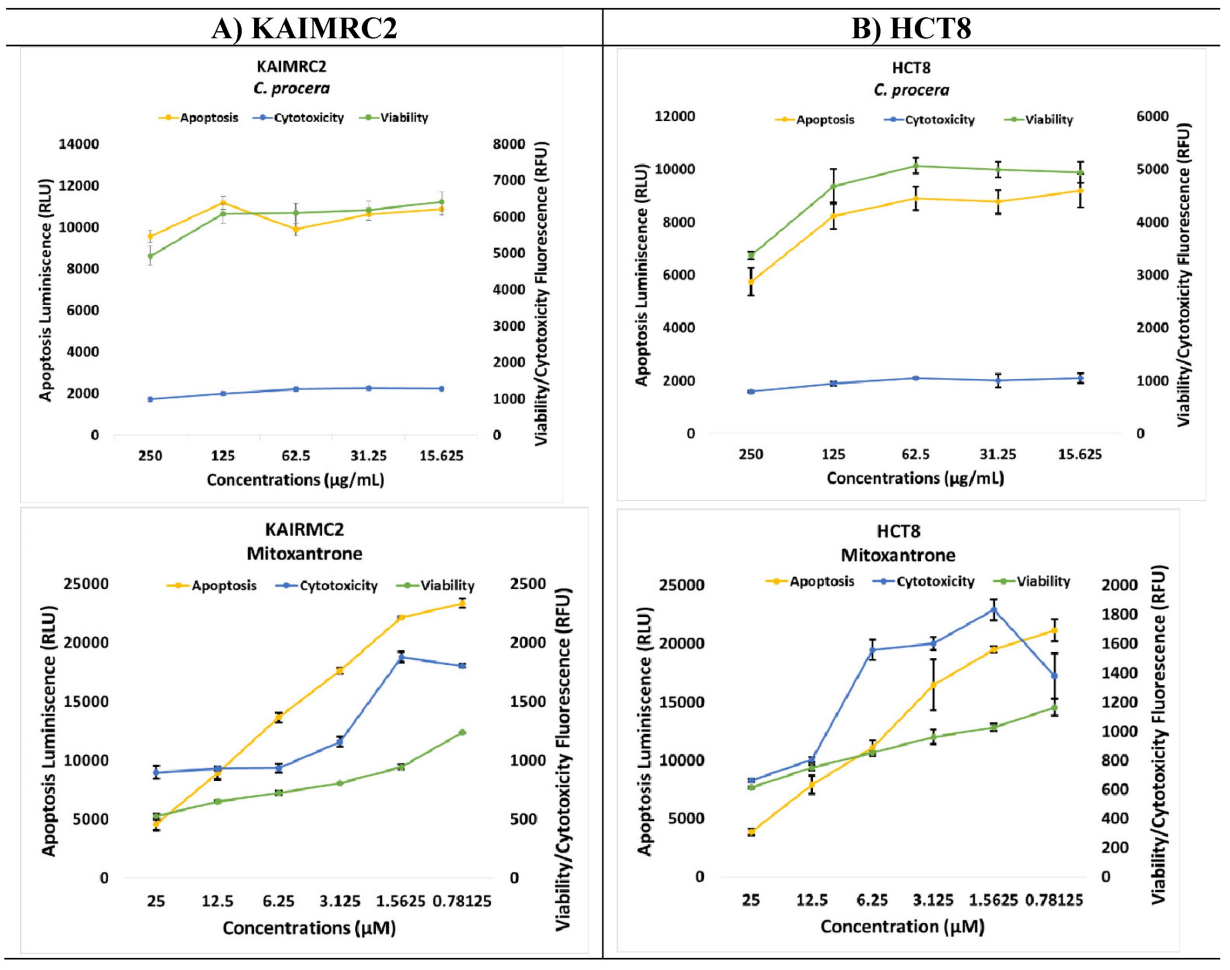
HCl Apoptosis Assay of A) Ethyl Acetate extract of *C. procera* and Mitoxantrone against KAIMRC2 and B) HCT8 cancer cell lines.

The ApoTox-Glo triplex assay was employed to assess the impact of the ethyl acetate extract from *C. procera* on the viability, cytotoxicity, and apoptosis of KAIMRC2 (**Figure 4A**) and HCT8 (**Figure 4B**) cell lines. The viability of both cell lines decreased as the concentration of the extract increased, with the most significant reduction observed at the highest concentration tested (250 µg/mL, p-value = 0.0119). Notably, there was no apparent dose-dependent effect on apoptosis in KAIMRC2 cells, whereas, in HCT8 cells, apoptosis seemed to decrease as the extract concentration increased. Moreover, both extracts showed no effects on cytotoxicity.

### Liquid Chromatography Quadrupole Time-Of-Flight Mass Spectrometry (LC-QTOF-MS)

**Figure 5:**
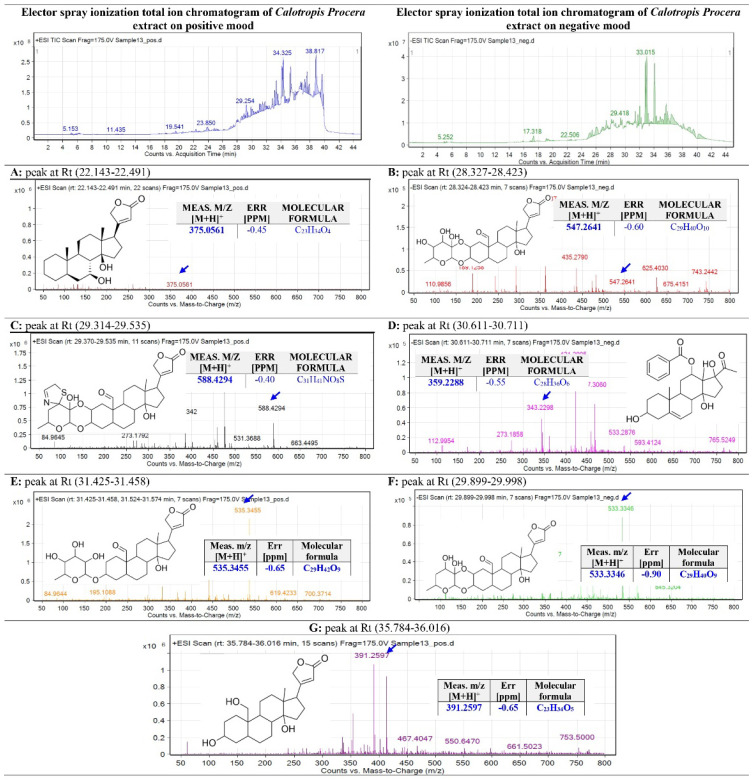
Base Peak Chromatogram of *C. procera* Ethyl Acetate Extract Was Extracted and Tentatively Identified Secondary Metabolites Are (A) proceragenin, (B) calotoxin, (C) uscharin, (D) calotropone, (E) gofrusid, (F) calactin, and (G) coroglaucigenin. Means *M/Z* Implies Measured *M/Z*.

The *C. procera* ethyl acetate extract was subjected to total ion current spectra (TIC) raw data, as shown in **Figure 5**. The data-analysis program Mass Hunter (Agilent Technologies) qualitative and quantitative analysis software was used to screen the below spectra. Chemical features were extracted from the LC-MS data using the Molecular Features Extraction (MFE) algorithm and the recursive analysis workflow. Features have been extracted by screening the detected nodes at various retention times per minute, with a minimum intensity of 6,000 counts, and aligned with previously detected compounds considering adducts ([M + H]^+^, [M + Na]^+^, [M + K]^+^ and ([M - H]^-^).

**Peak A:** The appeared m*/*z value at retention time (22.143-22.491) was correlated with the parent compound proceragenin 25 with m*/*z [M+H]^+^ 375.0561 daltons and a molecular formula of [C23H34O4]^+^, [M+H]^+^ m*/*z 374.25 in positive ion mode, and [M-H]^-^ with m*/*z 373.72 daltons in negative mode, indicating that the compound has a molecular weight of 374.51 g mol^-1^.

**Peak B:** The appeared m*/*z value at retention time (28.327-28.423) was correlated with the parent compound calotoxin 26 with m*/*z [M-H]^+^ 547.2641 daltons and a molecular formula of [C29H40O10]^+^, [M+H]^+^ m*/*z 548.26 in negative ion mode, and [M+H]^-^ with m*/*z 549.38 daltons in positive mode, indicating that the compound has a molecular weight of 548.62 g mol^-1^.

**Peak C:** The appeared m*/*z value at retention time (29.314-29.535) was correlated with the parent compound uscharin 26 with m*/*z [M+H]^+^ 588.4294 daltons and a molecular formula of [C31H41NO8S]^+^, [M-H]^+^ m*/*z 587.52 in positive ion mode, and [M+H]^-^ with m/z 587.72 daltons in negative mode, indicating that the compound has a molecular weight of 587.75 g mol^-1^.

**Peak D:** The appeared m*/*z value at retention time (30.611-30.711) was correlated with the parent compound calotropone 27 with m*/*z [M-H]^+^ 467.3060 daltons and a molecular formula of [C28H36O6]^+^, [M+H]^+^ m*/*z 468.65 in negative ion mode, and [M+H]^-^ with m*/*z 469.24 daltons in positive mode, indicating that the compound has a molecular weight of 468.75 g mol^-1^.

**Peak E:** The appeared m*/*z value at retention time (31.425-31.458) was correlated with the parent compound gofrusid 27 with m*/*z [M+H]^+^ 535.3455 daltons and a molecular formula of [C29H42O9]^+^, [M-H]^+^ m*/*z 536.45 in positive ion mode, and [M+H]^-^ with m/z 534.6 daltons in negative mode, indicating that the compound has a molecular weight of 534.6 g mol^-1^.

**Peak F**: The appeared m*/*z value at retention time (29.899-29.998) was correlated with the parent compound calactin 26 with m*/*z [M+H]^+^ 533.3346 daltons and a molecular formula of [C29H40O9]^+^, [M+H]^+^ m*/*z 532.564 in positive ion mode, and [M-H]^-^ with m/z 531.75 daltons in negative mode, indicating that the compound has a molecular weight of 532.85 g mol^-1^.

**Peak G:** The appeared m*/*z value at retention time (35.784-36.016) was correlated with the parent compound coroglaucigenin 26 with m*/*z [M+H]^+^ 391.2597 daltons and a molecular formula of [C22H32O5]^+^, [M+H]^+^ m*/*z 390.24 in positive ion mode, and [M-H]^-^ with m^/^z 390.01 daltons in negative mode, indicating that the compound has a molecular weight of 390.51 g mol^-1^.

### *In Silico* Computational Analysis

#### Prediction of Activity Spectra for the Identified Metabolites

The biological activities of a compound can be effectively predicted using computational approaches and the chemical structure of the molecule. One commonly used tool for this purpose is the PASS online web server. By analyzing the active-to-inactive ratio, the PASS web server can anticipate the biological activity (P_a_: P_i_) of a compound. To determine potential molecules with promising biological activity, a P_a_ value above 0.5 (P_a_ > 0.5) and a P_i_ value close to zero are considered favorable indicators.

In the case of the ethyl acetate extract of *C. procera*, all the metabolites present demonstrated P_a_ values greater than 80%. This suggests a high probability for these metabolites to exhibit active anti-cancer properties as summarized in **Table 2**. On the other hand, when assessing the prediction of anti-bacterial activity, calotoxin and calactin showed a 50% probability of being active as anti-bacterial agents. This implies that *C. procera* may have more prominent anti-cancer effects rather than anti-bacterial effects. These findings highlight the potential of *C. procera* as a valuable source of anti-cancer compounds.

#### Prediction of Bioactivity Scores

The Molinspiration webserver was utilized to predict the biological activity of the metabolites derived from *C. procera*. The compounds were evaluated for their potential as G-protein-coupled receptor (GPCR) ligands, ion channel modulators, kinase inhibitors, nuclear receptor inhibitors, protease inhibitors, and other enzyme inhibitors. The results showed that Proceragenin exhibited high biological activity in all targets except as a kinase inhibitor. It displayed the highest activity as a GPCR ligand and was the only compound predicted to be a potent ion channel modulator. All the metabolites demonstrated activity as nuclear receptor ligands and enzyme inhibitors. Calotropone showed the highest predicted activity as a nuclear receptor ligand, while calactin had the highest score as an enzyme inhibitor. However, none of the compounds were predicted to be active as kinase inhibitors. Furthermore, all the compounds, except calotropone and coroglaucigenin, were predicted to be active as protease inhibitors as summarized in **Table 3**.

**Figure 6:**
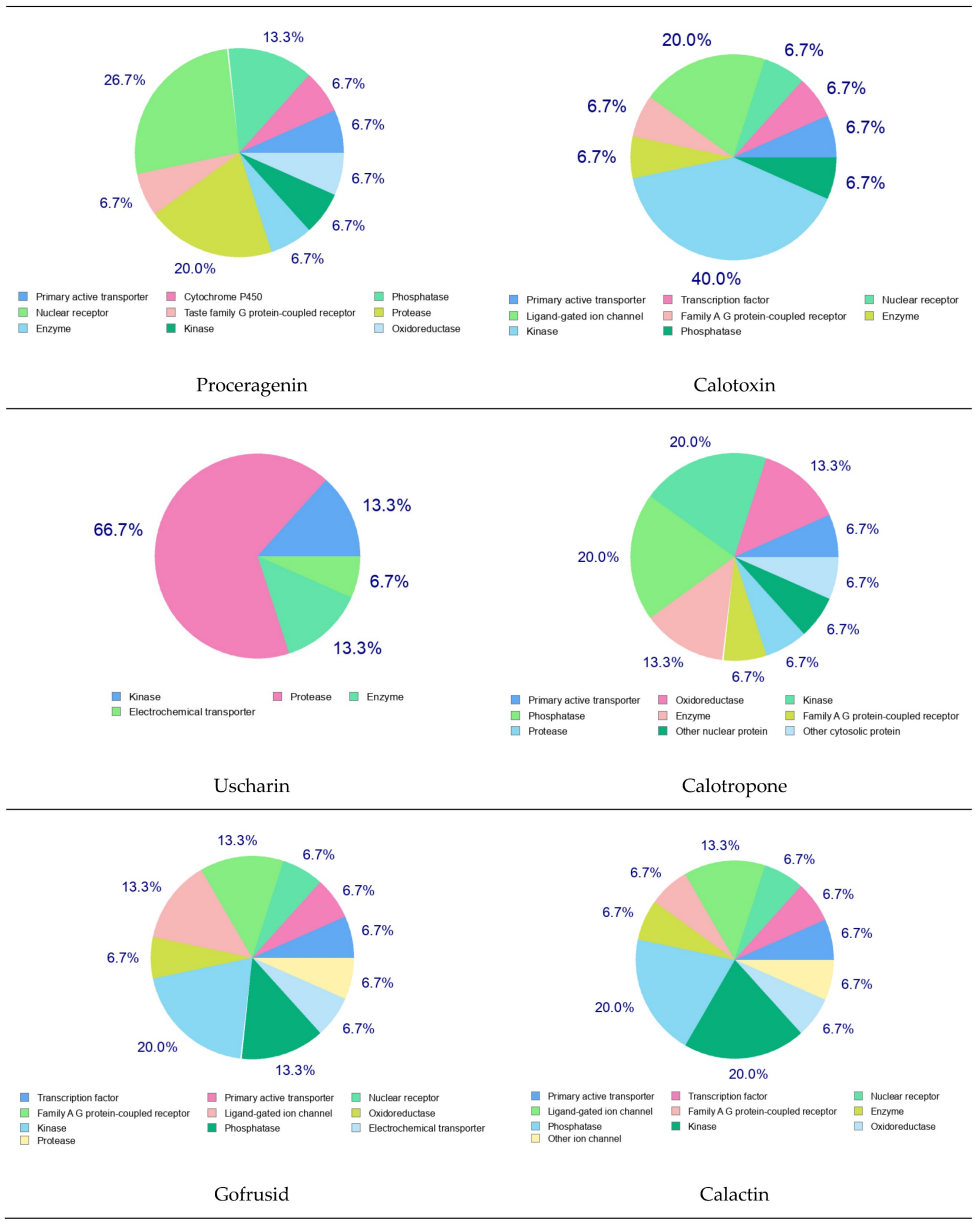

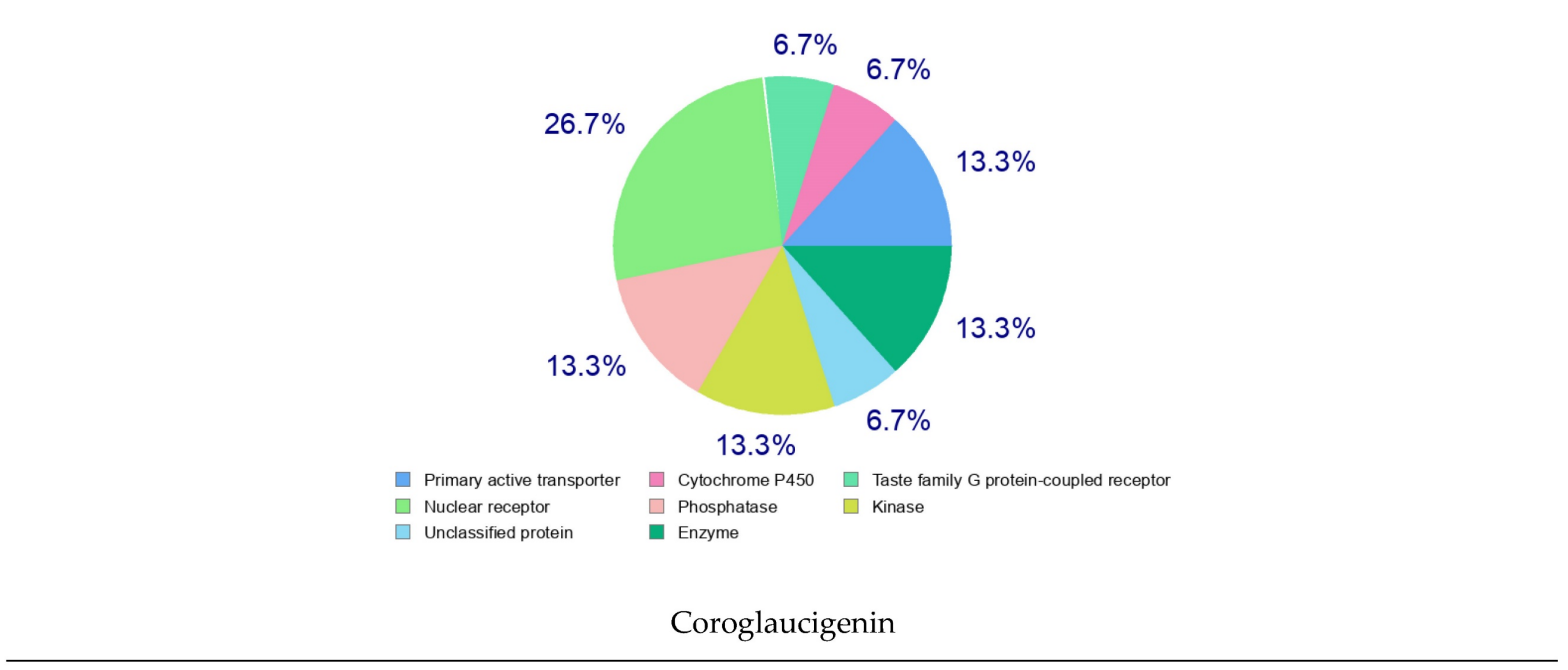
Swiss Target Prediction of Identified Metabolites of *C. procera* in Ethyl Acetate Extract.

Furthermore, the targets of the bioactive compounds identified in the *C. procera* extract were also determined using Swiss Target Prediction. The pie chart analysis of these metabolites revealed interesting findings. Proceragenin showed a 26.7% probability of being a nuclear receptor target and 20.0% as a protease target. Calotoxin, on the other hand, had a probable ligand-gated ion channel target of 20.0% and 40.0% for the kinase target. Uscharin exhibited a protease target of 66.7%. Calotropone showed kinase and phosphatase targets of 20.0%, while Gofrusid had a nuclear receptor target of 13.3% and a kinase target of 20.0%. Calactin's pie chart revealed a kinase and phosphatase target of 20.0%. Finally, Coroglaucigenin had a predicted nuclear receptor target of 26.7%, similar to Proceragenin as shown in **Figure 6**.

##### 4.6.3 Pharmacokinetic Parameters Evaluation of the Identified Metabolites of *C. procera* in Ethyl Acetate Extract

The pharmacokinetic characteristics of identified metabolites of *C. procera* in ethyl acetate were evaluated using the SwissADME website as summarized in **Table 4**. According to Lipinski's rule, which restricts molecular weight (MW) to less than 500, most of the active compounds derived from *C. procera* comply with this rule. However, calotoxin, uscharin, gofrusid, and calactin have molecular weights exceeding 500 g/mol. The number of hydrogen bond acceptors (HBA) and hydrogen bond donors (HBD) also play a role in drug-likeliness properties. All the identified metabolites of *C. procera* have the optimal number of HBD (0-5). However, calotoxin, uscharin, gofrusid, and calactin violate the drug's likeliness properties by having a high number of hydrogen bond acceptors (10, 9, 9, and 9, respectively).

To assess the hydrophobicity of the metabolites, three methods were used: iLOGP, XLOGP3, and MLOGP. The results indicate that all identified metabolites of *C. procera* in the ethyl acetate extract fall within the recommended range (-1 to 5) for Lipinski's rule of five (ROF) 29.

In terms of solubility, all identified metabolites of *C. procera* have log S values that do not exceed 6, which is within the acceptable boundaries for oral absorption. Proceragenin, calotropone, calactin, and coroglaucigenin are predicted to be well-absorbed from the gastrointestinal tract. Additionally, Proceragenin is the only metabolite that can cross the blood-brain barrier (BBB).

Regarding potential interactions with cytochrome P450 enzymes, all identified metabolites of *C. procera*, except for calotropone, do not inhibit these enzymes. This suggests a low likelihood of drug-herbal interactions. However, calotoxin, uscharin, gofrusid, and calactin exhibit molecular weights above 500 g/mol, which may affect their oral activity. Proceragenin, calotropone, and coroglaucigenin do not violate Lipinski's rule of five and are deemed suitable for oral bioavailability.

#### Assessment of Oral Bioavailability

**Figure 7:**
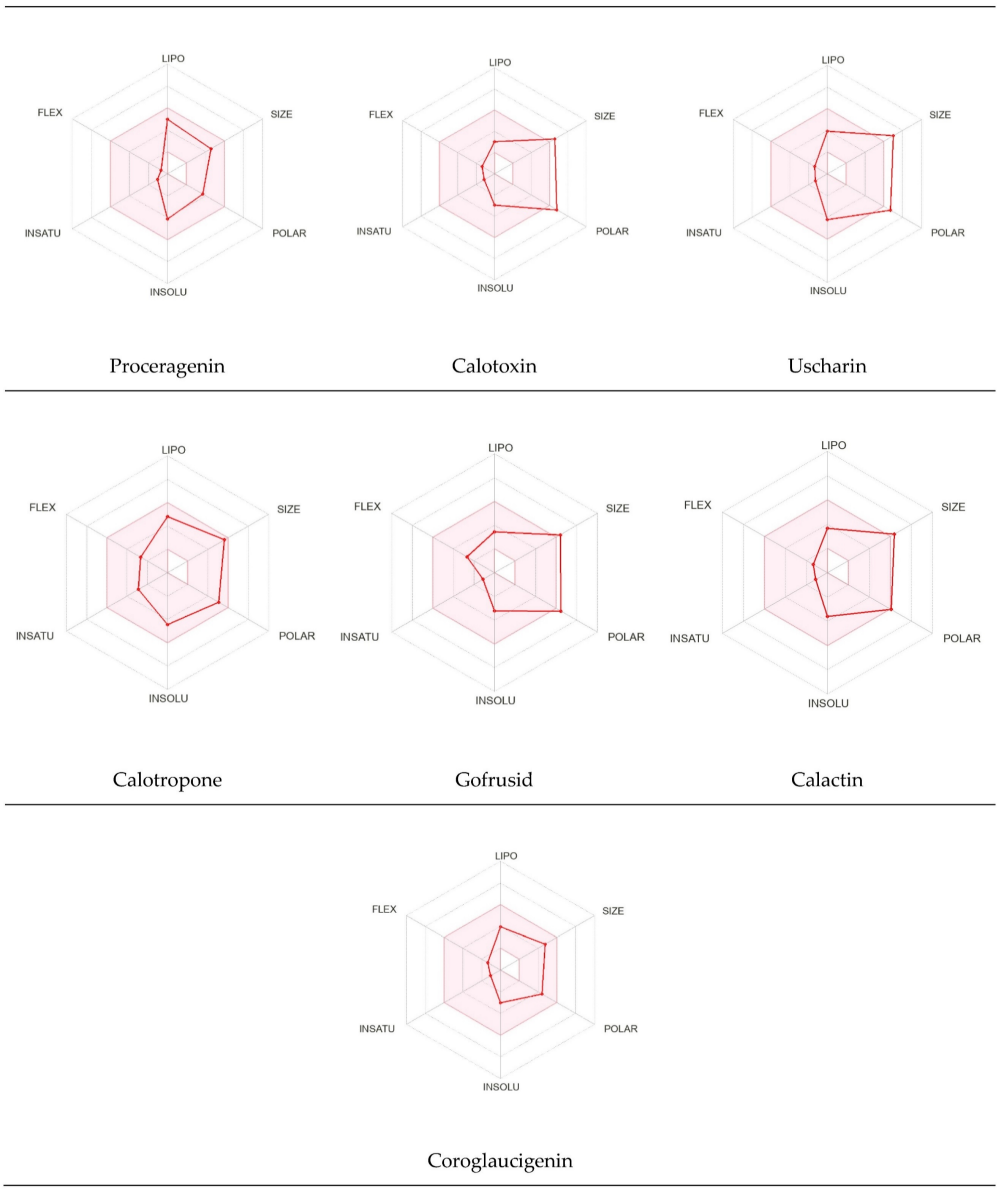
Radar Charts for Oral Bioavailability of the Identified Metabolites of *C. procera* in Ethyl Acetate Extract.

The bioavailability radar was employed to assess the polarity (POLAR), solubility (INSOLU), lipophilicity (LIPO), flexibility (FLEX), saturation (INSATU), and size (SIZE) of the identified metabolites 28-30. **Figure 7** depicts the predicted properties (red line) of the evaluated molecules, with the recommended range for oral activity represented by the pink-shaded region. Consequently, our findings indicate that only three metabolites, namely Proceragenin, calotropone, and coroglaucigenin, are anticipated to exhibit oral bioavailability.

#### 4.6.5 Organ and End-Point Toxicity Prediction

According to the computational analysis and toxicity evaluation of the identified metabolites of *C. procera*, it was found that all the metabolites were immunotoxic and cytotoxic, except for Proceragenin and calotropone, which did not exhibit cytotoxicity. It is crucial to assess whether the observed immunotoxic and cytotoxic effects are balanced by specific mechanisms that selectively target cancer cells. Understanding how the extract operates and its impact on cancer cells compared to normal cells is critical for evaluating its potential as an anticancer therapy. Additionally, evaluating the extract's therapeutic index, which compares its effectiveness in eradicating cancer cells to its toxicity to normal cells, is vital for determining its suitability as an anticancer treatment. A narrow therapeutic index, characterized by significant toxicity to normal cells, could limit the clinical applicability of the extract. Exploring formulation strategies to enhance the extract's specificity towards cancer cells while minimizing off-target effects could significantly improve its potential as an anticancer therapy. These considerations can be evaluated and addressed for future assessment, which offers valuable directions for future research and evaluation. Furthermore, none of the metabolites showed hepatotoxicity, carcinogenicity, or mutagenicity, as indicated in **Table 5**.

#### Cardiac Toxicity Prediction

The blockage of the human ether-a-go-go-related gene (hERG) K^+^ channels is strongly associated with lethal cardiac arrhythmias and premature death 31. To assess cardiotoxicity, we utilized the pred-hERG website, which provides free accessibility to the webserver. In **Table 6**, the probability map illustrates the following: green atoms represent hERG blocking, pink atoms indicate reduced hERG blockage and gray atoms signify the location of a split between positive (green) and negative (pink) contributions 23. Our findings indicate that Proceragenin, calotoxin, and coroglaucigenin are predicted to be non-cardiotoxic with a 50% confidence level. Conversely, uscharin, calotropone, gofrusid, and calactin are all projected to be cardiotoxic with a 50% certainty level.

#### Nuclear Receptor Binding Prediction of Ethyl Acetate *C. procera* Extract

The Endocrine Disruptome, an open-source prediction tool, is used to assess the binding of molecules to multiple nuclear receptors, including the androgen receptor (AR), estrogen receptors (ERα and ERβ), glucocorticoid receptor (GR), liver X receptors (LXRα and LXRβ), mineralocorticoid receptor (MR), peroxisome proliferator-activated receptors (PPARα, PPARβ/δ, and PPARγ), progesterone receptor (PR), retinoid X receptor α (RXRα), and thyroid receptors (TRα and TRβ). Our results for the binding of *C. procera* metabolites to these nuclear receptors are summarized in **Table 7**. The red color indicates a high likelihood of binding, whereas green indicates a low likelihood of binding. None of the metabolites displayed a high likelihood of binding to any of the nuclear receptors.

## Discussion

Our study focused on the therapeutic potential of two local herbs, *C. procera* and *R. stricta*, from the *Apocynaceae* family. We aimed to investigate their antibacterial and anticancer effects, identify their active metabolites, and predict their pharmacodynamic, pharmacokinetic, and toxicity profiles. In terms of antibacterial activity, our study confirmed that *C. procera* and *R. stricta* are valuable sources of phytochemicals with antibacterial properties. Specifically, three solvent extracts from *R. stricta* leaves demonstrated effectiveness against *E. coli* and methicillin-resistant *S. aureus* (MRSA). These findings align with a recent study that highlighted the antibacterial activity of the ethanolic extract of *C. procera* against *S. aureus* and *E. coli* 32.

In terms of anti-cancer activity, the ethyl acetate extract of *C. procera* demonstrated potent cytotoxic effects on human hepatocellular carcinoma and colorectal cancer cell lines. These results prompted a focused exploration of *C. procera* extracts due to their comparatively higher potency, thus guiding the direction of the subsequent investigations. Further tests on the KAIMRC2 cell line, a highly proliferative breast cancer cell line with stem cell-like characteristics, revealed a strong cytotoxic effect. Our results suggest that the distinct protein and gene expression profiles of KAIMRC2 may contribute to the enhanced cytotoxic effect of the extract.

However, the results of the ApoTox triplex Glo assay did not align with the MTT assay and HCI study, possibly due to several factors. Firstly, at these concentrations, the herbal extracts might have reached a saturation point in their cytotoxic or cytostatic effects, leading to minimal additional impact on cell viability despite further increases in concentration. Additionally, the discrepancy in response could stem from the herbal extracts exerting diverse effects at different concentration ranges, with higher concentrations triggering different cellular responses, potentially leading to a plateau in cytotoxicity and viability changes. Moreover, cellular adaptation to the presence of the herbal extracts over the 48-hour duration may have stabilized cytotoxic effects, regardless of the proximity to the IC_50_ values. Lastly, the assay's sensitivity at these concentrations might not be adequate to detect subtle changes in cellular response, contributing to the consistent data despite varying concentrations. These factors collectively contribute to the observed lack of significant changes in cytotoxicity and viability data at concentrations near the IC_50_ values obtained from the MTT assay at 48 hours 19, 33, 34.

Ethyl acetate, the solvent used for extracting medicinal plants, is known for its moderate polarity and ability to interact with charged polar and non-polar biological compounds. It has low toxicity and reversible toxic effects on liver and kidney tissues, as highlighted by a previous study 35. Using ethyl acetate, we have identified seven active metabolites from *C. procera*, including proceragenin 25, calotoxin, uscharin 26, calotropone, gofrusid 27, calactin, and coroglaucigenin 26. Some of these metabolites, classified as cardenolides, are plant steroids that often exhibit cardiac activities and can be toxic to the heart 36. However, in the current study, three out of the seven metabolites (coroglaucigenin, calotoxin, and proceragenin) were predicted to be non-cardiotoxic. This suggests that these compounds may be safer options for further research and development.

Many of these compounds derived from *C. procera* have shown cytotoxic effects against various cancer cells, including lung cancer and colorectal cancer 37. Additionally, Proceragenin, a compound from *Calotropis procera*, has strong antibacterial activities against Gram-positive and Gram-negative bacteria 25. Coroglaucigenin has been found to induce senescence and autophagy in colorectal cancer cells by inhibiting CD4K and Akt pathways 38. In a study on human lung cancer cells, coroglaucigenin increased radiosensitivity by preventing the up-regulation of antioxidant molecules 39. Calotoxin and calactin were found to target interleukin-2 inducible T cell kinase (ITK), suggesting their potential as lead molecules for creating novel ITK inhibitors with therapeutic applications as immune suppressants and anticancer medications 40. Cardenolides extracted from *Calotropis gigantea*, such as uscharin, calactin, and calotropin, have been shown to bind directly to RORγt, the main transcription factor of Th17 lymphocyte differentiation, and suppress Th17 differentiation, making them potential compounds for treating autoimmune disorders 40.

Although natural products show promise for cancer treatment, clinical concerns have been raised due to the lack of sufficient evidence regarding their efficacy, molecular targets, physicochemical properties, and safety 41. In order to identify potential bioactive metabolites derived from *C. procera*, computational approaches utilizing tools such as PASS online, Molinspiration, SwissADME, and ProTox-II were employed 41. These approaches aim to predict anti-cancer activity, molecular targets, physicochemical properties, and safety evaluation of the identified metabolites 41. The computational evaluation revealed that the metabolites of *C. procera* in ethyl acetate exhibited a promising anti-cancer activity of over 80%. Furthermore, a study utilized a reverse screening technique to identify possible anticancer targets of calactin, calotropin, and calotoxin 42. It was found that these metabolites have the potential to target Interleukin-2 inducible T cell kinase (ITK) 42. This suggests that these substances could serve as lead molecules for the development of ITK inhibitors, which can have therapeutic applications as immune suppressants and anticancer medications 42.

According to the Molinspiration Webserver, the identified metabolites of *C. procera* exhibited activity as nuclear receptor ligands and enzyme inhibitors, as indicated by their high bioactivity scores in computational predictions. However, metabolites did not demonstrate significant activity as kinase inhibitors. This is significant because nuclear receptor ligands have shown promise in hormonally dependent cancers, where malfunctioning nuclear receptors are often observed and targeted. The identification of these metabolites suggests their potential as therapeutic targets for cancer patients, especially those resistant to current medications targeting hormone nuclear receptors 43. The SwissTarget Prediction web server was also utilized to gain insights into the molecular processes and potential side effects of the identified metabolites. Once again, it confirmed their activity as nuclear receptor ligands and enzyme inhibitors, while not predicting high activity as kinase inhibitors. Such information is valuable for understanding the bioactivity and off-target effects of known molecules.

Furthermore, the lipophilicity of the metabolites, as estimated by the log p-value, was within the recommended range according to Lipinski's rule of five. This parameter is crucial for assessing the potency, distribution, and elimination of drugs in the body 21, 28. The permeability of substances through the skin, known as skin permeability, is measured by log Kp. The metabolites of *C. procera* were found to be less skin permeant based on their more negative log Kp values. Additionally, the presence of the blood-brain barrier (BBB) poses challenges in developing treatments for brain tumors and limited metabolite that demonstrates the permeability through BBB suggesting more peripheral application for these metabolites 44. Moreover, the metabolites were predicted to be orally absorbable, and soluble which could support the oral activity.

Concerns regarding herb-drug interactions arise from the fact that active ingredients in herbs can significantly alter the pharmacokinetic and pharmacodynamic characteristics of drugs. One proposed mechanism for such interactions is the inhibition or activation of cytochrome P450 (CYP450) enzymes. Fortunately, the identified metabolites did not inhibit most CYP450 enzymes, except for calotropone, which primarily inhibited the CYP3A4 enzyme. This suggests a low potential for drug-herb interactions 45, 46, 47.

Furthermore, the identified metabolites of *C. procera* from the Ethyl Acetate extract were evaluated for toxicity using the WebProtox II Webserver 22. The results showed that all the metabolites except for Proceragenin and calotropone exhibited immunologic and cytotoxic properties. Additionally, none of the metabolites showed hepatotoxicity, carcinogenicity, or mutagenicity. However, it was predicted that the hERG K^+^ channel blockade by some metabolites could potentially lead to QT prolongation and fetal arrhythmias. On the other hand, the metabolites Proceragenin, calotoxin, and coroglaucigenin were expected to be non-cardiotoxic, with a 50% degree of confidence. Conversely, uscharin, calotropone, gofrusid, and calcactin were predicted to be cardiotoxic with a 50% certainty level.

Moreover, an open-source prediction program called Endocrine Disruptome revealed that certain metabolites of *C. procera* could potentially interact with various nuclear receptors 24. These receptors include thyroid receptors TRα and TRβ, androgen receptor (AR), pregnane X receptor (PXR), peroxisome proliferator-activated receptors (PPARα, PPARγ), estrogen receptors, constitutive androstane receptor (CAR), estrogen-related receptor (ERR), retinoid X receptors (RXRα, RXRβ, and RXRγ), progesterone receptor (PR), mineralocorticoid receptor (MR), glucocorticoid receptor (GR), retinoic acid receptors (RARα, RARβ, and RARγ), farnesoid X receptor (FXR), and liver X receptors. Interestingly, none of the evaluated metabolites demonstrated interactions with nuclear receptors suggesting the lower potential for adverse effects that could result from the activation of various nuclear receptors. It is worth mentioning that there was a discrepancy between the Molinspiration webserver's prediction of metabolites from *C. procera* as nuclear receptor ligands and the Endocrine Disruptome's demonstration of a low likelihood of binding to nuclear receptors which could be attributed to differences in prediction methodologies, algorithms, and molecular descriptors used by the two platforms leading to varying outcomes in predicting the metabolites' activity as nuclear receptor ligands. The differing priorities of the Molinspiration webserver and Endocrine Disruptome in evaluating nuclear receptor binding, potentially contribute to the observed inconsistency. The Endocrine Disruptome's focused assessment of 14 specific human nuclear receptors possibly resulted in different outcomes compared to the broader assessment by the Molinspiration webserver. The influence of crystal structures of nuclear receptors used by the Endocrine Disruptome for docking studies could lead to discrepancies if metabolite interactions with specific receptors were not adequately considered.

## Conclusion

In conclusion, our study highlights the potential activity of *R. stricta* and *C. procera* as valuable anti-bacterial agents against *E. coli* and *S. aureus*. Moreover, we have identified ethyl acetate as an optimal solvent for producing a cytotoxic *C. procera* extract that shows promise in combating various types of cancers. Additionally, the secondary metabolites derived from *C. procera*, namely Proceragenin, calotropone, and coroglaucigenin, hold potential as orally bioavailable lead compounds for the development of a new class of anticancer agents targeting nuclear receptors. However, it is important to note that further experiments are required to assess the individual *in vitro* biological activities, as well as the pharmacokinetic and toxicity profiles of these compounds. These crucial investigations will provide a deeper understanding of their therapeutic potential. Overall, our findings provide a stepping stone toward exploring the future therapeutic applications of *C. procera* and *R. stricta*. Additionally, this research can guide scientists in optimizing experiments for early drug discovery, harnessing the pharmacological benefits of these plants. By continuing to delve into the potential of these pharmacologically active plants, we can pave the way for novel therapeutic interventions in the future.

## Figures and Tables

**Table 1:** The IC_50_ (µg\mL) of *C. procera* and *R. stricta* using Multiple Solvents Against Several Cancer Cell Lines using MTT Assay.

Name	HepG2 IC_50_ (µg\mL)	HepG2 Standard Deviation of the Residuals (Sy.x)*	HCT8 IC_50_ (µg\mL)	HCT8 Standard Deviation of the Residuals (Sy.x)*
CP in ethanol	790.9	103.9	464.6	104.8
RS in ethanol	744.8	101.2	609.4	76.4
CP in chloroform	5245	88.8	8677	137.7
RS in chloroform	2404	92.7	6500	95.8
CP in ethyl acetate	194.0	118.1	112.6	148.0
RS in ethyl acetate	2109	106.9	903.5	87.0
CP in water	3289	43.8	246.9	91.1
RS in water	1265	179.1	1423	180.2
Mitoxantrone	0.2970	7.842	0.09014	7.707
Name	MDA-MB-231 IC_50_ (µg\mL)	MDA-MB-231 Sy.x	KAIMRC2 IC_50_ (µg\mL)	KAIMRC2 Sy.x
CP in ethyl acetate	207.4	6.781	54.37	8.543
Mitoxantrone	0.6228	9.638	0.4215	7.451

* The purpose of Sy.x is to provide a robust assessment of the standard deviation of the residuals, taking into consideration the number of parameters fit by regression, and enabling a more accurate evaluation of the model's fit to the data.

**Table 2:** Prediction of Activity Spectra for the Identified Metabolites of *C. procera* in Ethyl Acetate Using PASS Online Website.

Biological Activities for Metabolites	Anti-cancer Activity			Anti-bacterial Activity	
	P_a_	P_i_		P_a_	P_i_
**Proceragenin**	0.831	0.008		0.188	0.129
**Calotoxin**	0.948	0.004		0.527	0.014
**Uscharin**	0.955	0.004		0.299	0.061
**Calotropone**	0.811	0.010		NA	NA
**Gofrusid**	0.898	0.005		0.485	0.018
**Calactin**	0.950	0.004		0.526	0.014
**Coroglaucigenin**	0.882	0.005		0.189	0.128

P_a_: Probability of being active. P_i_: Probability of being inactive. NA: not active.

**Table 3:** Molecular Target Prediction of Identified Metabolites of *C. procera* in Ethyl Acetate Extract.

Metabolites	GPCR Ligand	Ion Channel Modulator	Kinase Inhibitor	Nuclear receptor Ligand	Protease Inhibitor	Enzyme Inhibitor
Proceragenin	0.15	0.15	-0.38	0.51	0.03	0.74
Calotoxin	0.04	-0.11	-0.34	0.35	0.07	0.80
Uscharin	-0.08	-0.33	-0.54	0.15	0.03	0.67
Calotropone	-0.09	-0.15	-0.59	0.55	-0.13	0.41
Gofrusid	0.08	-0.04	-0.32	0.22	0.12	0.73
Calactin	0.07	-0.01	-0.29	0.52	0.12	0.88
Coroglaucigenin	0.11	-0.02	-0.35	0.57	-0.02	0.80

**Table 4:** Pharmacokinetic Prediction of Identified Metabolites of *C. procera* in Ethyl Acetate Extract.

Properties	Parameters	Proceragenin	Calotoxin	Uscharin	Calotropone	Gofrusid	Calactin	Coroglaucigenin
Physicochemical Properties	MW (g/mol)	374.51	548.62	587.72	468.58	534.64	532.62	390.51
	HBA	4	10	9	6	9	9	5
	HBD	2	4	2	3	4	3	3
Lipophilicity Log Po/w	iLOGP	3.19	2.98	3.38	3.61	2.96	2.93	3.00
	XLOGP3	3.19	-0.24	1.36	2.92	0.51	0.93	1.42
	MLOGP	3.56	0.72	1.78	2.85	1.08	1.49	2.73
Absorption	Water solubility	-3.46 (Soluble)	-1.52 (Soluble)	-3.55 (Soluble)	-4.83 (Moderately soluble)	-1.82 (Soluble)	-2.35 (Soluble)	-2.90 (Soluble)
	GI	High	Low	Low	High	Low	High	High
	Log Kp (skin permeation) cm/s	-6.32	-9.82	-8.92	-7.09	-9.20	-8.89	-7.67
Distribution	BBB permeant	Yes	No	No	No	No	No	No
Metabolism	CYP1A2 inhibitor	No	No	No	No	No	No	No
	CYP2C19 inhibitor	No	No	No	No	No	No	No
	CYP2C9 inhibitor	No	No	No	No	No	No	No
	CYP2D6 inhibitor	No	No	No	No	No	No	No
	CYP3A4 inhibitor	No	No	No	Yes	No	No	No
Drug likeness	Lipinski	Yes; 0 violation	Yes; 1 violation: MW>500	Yes; 1 violation: MW>500	Yes; 0 violation	Yes; 1 violation: MW>500	Yes; 1 violation: MW>500	Yes; 0 violation

MW: Molecular weight. HBA: hydrogen-bonding acceptor. HBD: hydrogen-bonding donor.

**Table 5:** Toxicity Evaluation for the Identified Metabolites of *C. procera* Ethyl Acetate Extract Using Protox II Webserver.

Metabolite Number	Classification					
	Organ Toxicity (%Probability)		Toxicity Endpoint (% Probability)			
	Hepatotoxicity		Carcinogenicity	Immunotoxicity	Mutagenicity	Cytotoxicity
Proceragenin	Inactive (0.82)		Inactive (0.70)	Active (0.99)	Inactive (0.78)	Inactive (0.51)
Calotoxin	Inactive (0.95)		Inactive (0.74)	Active (0.99)	Inactive (0.77)	Active (0.97)
Uscharin	Inactive (0.82)		Inactive (0.58)	Active (0.99)	Inactive (0.67)	Active (0.76)
Calotropone	Inactive (0.83)		Inactive (0.53)	Active (0.98)	Inactive (0.88)	Inactive (0.70)
Gofrusid	Inactive (0.97)		Inactive (0.75)	Active (0.99)	Inactive (0.78)	Active (0.92)
Calactin	Inactive (0.94)		Inactive (0.69)	Active (0.99)	Inactive (0.80)	Active (0.99)
Coroglaucigenin	Inactive (0.85)		Inactive (0.74)	Active (0.99)	Inactive (0.76)	Active (0.79)

**Table 6:** Prediction of Cardiotoxicity of the Identified Metabolites of *C. procera* in Ethyl Acetate Using Pred-hERG Webserver.

Metabolite Name	Prediction / Potency	Confidence	Probability Map
Proceragenin	Non-cardiotoxic (-)	50%	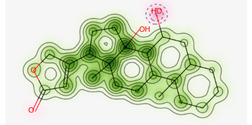
Calotoxin	Non-cardiotoxic (-)	50%	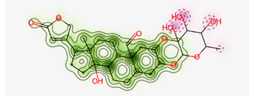
Uscharin	Potential cardiotoxic (+)	50%	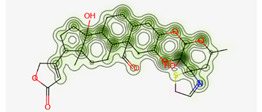
Calotropone	Potential cardiotoxic (+)	50%	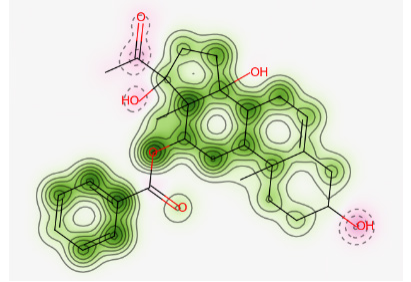
Gofrusid	Potential cardiotoxic (+)	50%	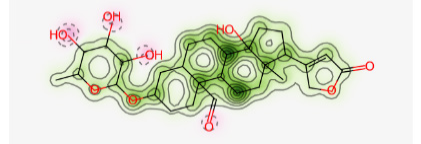
Calactin	Potential cardiotoxic (+)	50%	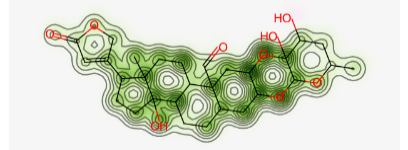
Coroglaucigenin	Non-cardiotoxic (-)	50%	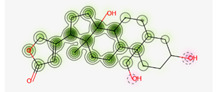

**Table 7:** Endocrine Disruptome Predictions of Identified Metabolites of *C. procera* in Ethyl Acetate Extract.

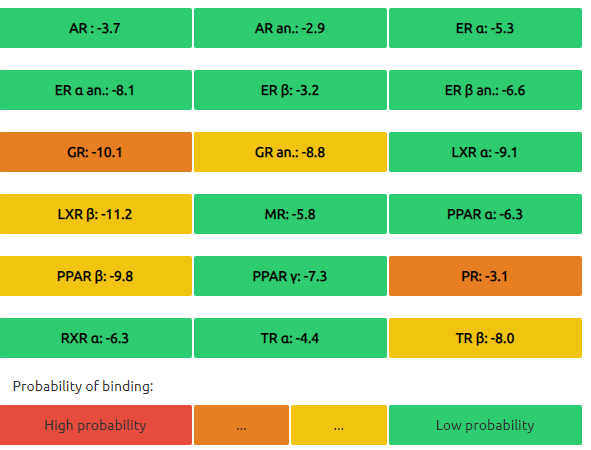	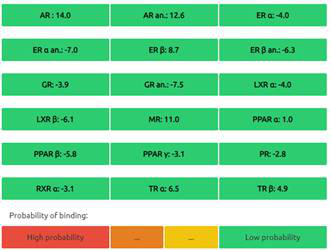
**Proceragenin**	**Calotoxin**
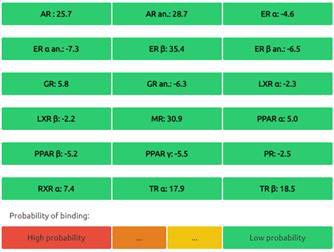	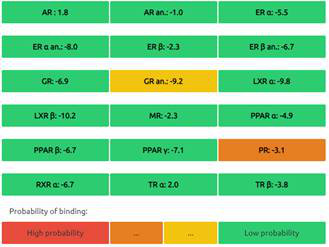
**Uscharin**	**Calotropone**
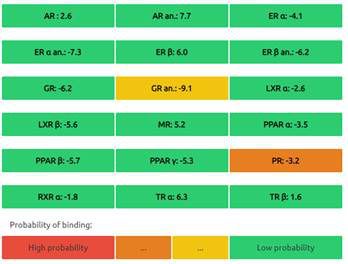	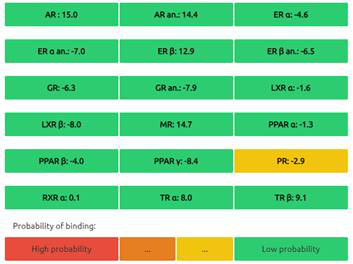
**Gofrusid**	**Calactin**
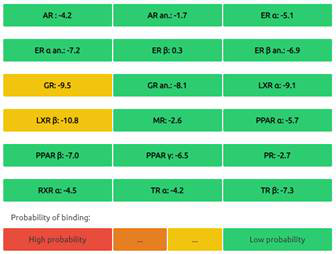	
**Coroglaucigenin**	

## Abbreviations

*S. aureus*: *Staphylococcus aureus*
*E. coli*: *Escherichia coli*
HCT8: Human ileocecal adenocarcinoma cell line
HepG2: Human liver cancer cell line
MDA-MB-231: Triple-negative breast cancer
KAIMRC2: King Abdullah international medical research center 1 cell line
IC_50_: Half-maximal inhibitory concentration
Pa: Probability of being active
Pi: Probability of being inactive
NA: Not active
GPCR: G-protein-coupled receptors
MW: Molecular weight
HBA: Hydrogen-bonding-acceptor
HBD: Hydrogen-bonding-donor
Log P: Lipophilicity
Log Kp: skin permeation
BBB: Blood-brain barrier
POLAR: Polarity
INSOLU: Solubility
LIPO: Lipophilicity
FLEX: Flexibility
INSATU: Saturation
SIZE: size
AR: Androgen receptor
ER: Estrogen receptor
PR: Progesterone receptor
GR: Glucocorticoid receptor
MR: Mineralocorticoid receptor
PXR: Pregnane X receptor
PPAR: Peroxisome proliferator-activated receptors
RXR: Retinoid X receptor
RAR: Retinoic acid receptors
TR: Thyroid receptor
FXR: Farnesoid X receptor
LXR: Liver X receptors
AST: Antimicrobial Susceptibility Testing
HCI: High content imagining
